# Case Report: Managing a giant, high-grade carotid body tumor in a resource-limited setting

**DOI:** 10.12688/f1000research.12726.1

**Published:** 2017-10-04

**Authors:** Sunil Munakomi, Samrita Chaudhary, Iype Cherian

**Affiliations:** 1 Department of Neurosurgery, Nobel Teaching Hospital, Biratnagar, Nepal

**Keywords:** giant, carotid body, tumor

## Abstract

Herein we report the management of a giant, high-grade and vascular carotid body tumor in a young woman. She presented with slowly progressive neck swelling. Vascular imaging revealed a left-sided, high-grade giant carotid body tumor (> 8cm).  The tumor was completely excised by caudocranial subadventitial dissection. Histology of the tumor revealed a characteristic Zellballen pattern of the lesion, suggestive of a paraganglioma. The patient made an uneventful recovery. We also discuss newer insights regarding the management of such highly vascular lesions.

## Introduction

Giant carotid body tumors are rare. Management of such highly vascular lesions, which completely encase the major carotid artery along with its branches, is even more challenging. We report the management strategy for a similar case, where caudocranial subadventitial dissection completely excised this tumor, one of the largest reported in the literature ^1, 2^.

## Case report

A 22-year-old woman presented to the Neurosurgery clinic in Nobel Teaching Hospital with a history of slow progressive but painless swelling over her left neck, ongoing for the last 6 months. She was also experiencing some difficulty while swallowing. She denied history of trauma, any episodes suggestive of transient ischemic attacks or paroxysmal episodes of severe headache, flushing or chest pain. She did not have major surgery or medical illnesses in the past, or any significant family history.

**Figure 1.**
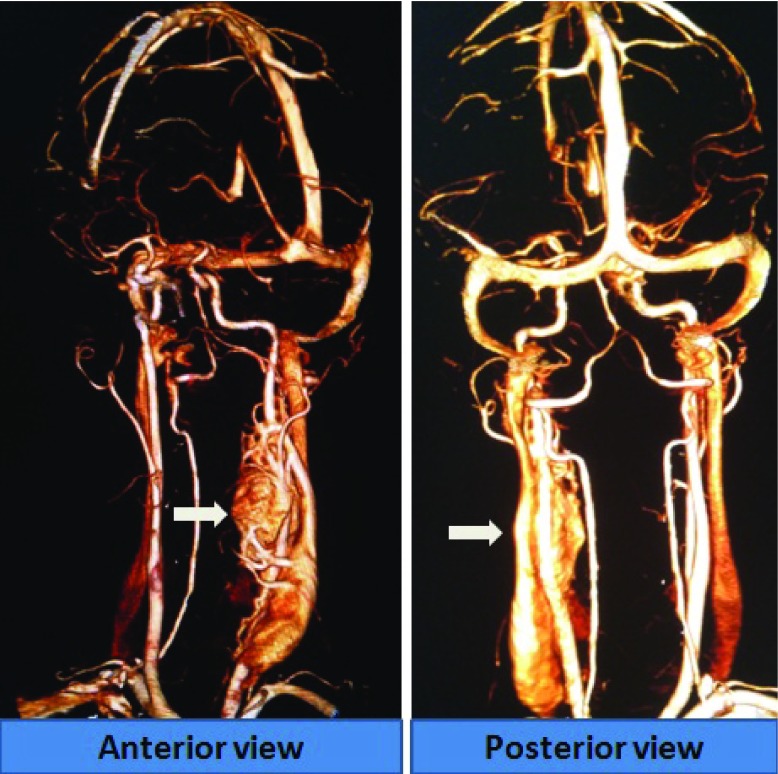
Carotid angiography image revealing a giant highly vascular, left-sided carotid body tumor.

**Figure 2.**
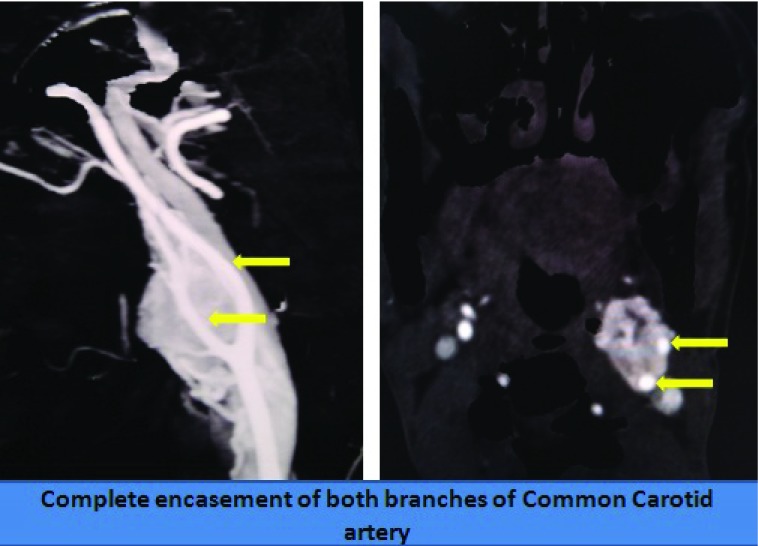
Angiographic image revealing encasement of the common carotid as well as both of its main branches by the tumor.

**Figure 3.**
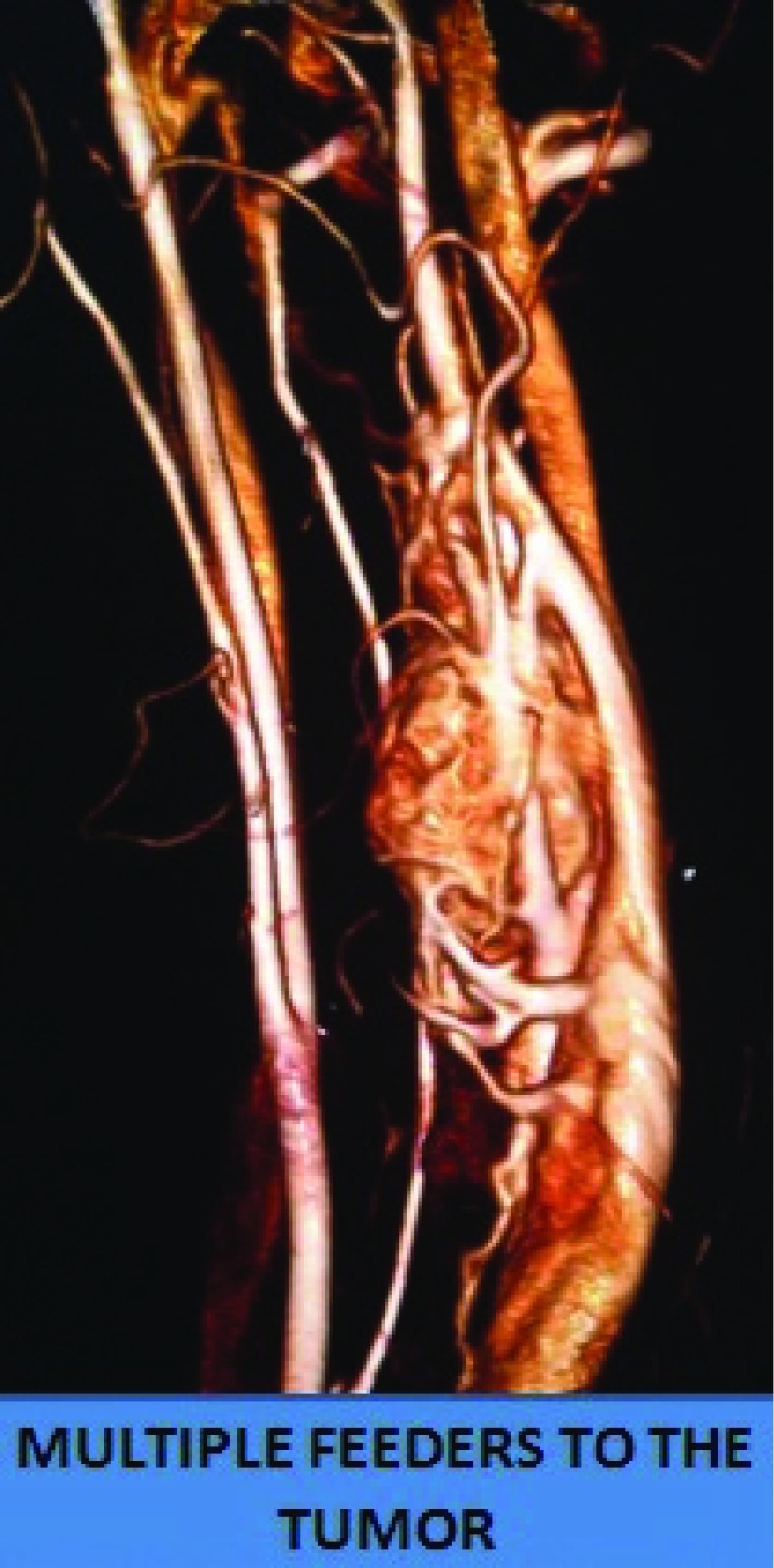
Tumor having vascular supply from both the external and internal carotid arteries.

Local examination revealed a pulsatile swelling on her left neck. There was no audible bruit over the swelling. The patient then underwent a CT angiography, which revealed a well-defined large (> 8 cm) heterogeneous and hyperdense soft tissue lesion; showing intense arterial enhancement at the level of carotid bifurcation ( Figure 1). It was causing significant compression and displacement of a long segment of the internal carotid artery (ICA) and external carotid artery (ECA), encasing a broad area of the ICA and ECA ( Shamblin Grade 3) ( Figure 2). The lesion was getting vascular supply from both the ICA and ECA branches ( Figure 3). These findings were all highly suggestive of a carotid body tumor.

Ultrasound imaging of the abdomen to assess adrenal glands was normal. 24-hour urine vanillylmandelic acid (VMA) and plasma metanephrines were within normal range.

**Figure 4.**
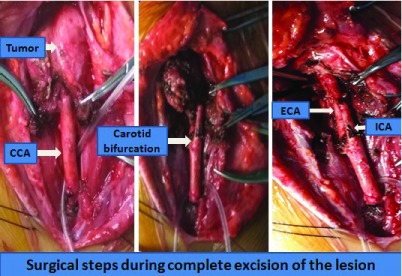
Surgical steps carried out during caudocranial subadventitial dissection.

**Figure 5.**
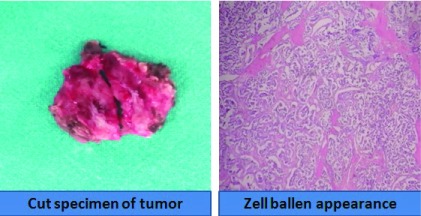
Cut specimen of the excised tumor, along with the characteristic Zellballen pattern in the histology.

The patient was thoroughly counseled regarding her condition and the need for operative management. The risks associated with the surgery, including intra-operative uncontrolled bleeding, lower cranial nerve palsy, ischemic stroke and even death, were detailed. Anaesthetic precautionary measures were implemented to reduce the risk of hypertensive crisis (during tumor manipulation) and hypotensive episodes (following tumor removal), by administering antihypertensive drugs and fluid support, respectively. After ensuring temporary carotid control with vascular loops, sub-adventitial dissection of the lesion was carried out starting from the common carotid caudally and then progressing cranially towards the bifurcation and its branches ( Figure 4). The major vascular supplies were sequentially isolated, ligated and divided. A venous graft from the long saphenous vein was prepared for repair in case of inadvertent tears within the carotid or any of its branches. The internal jugular vein and the vagus nerve within the carotid sheath, and the hypoglossal nerve were all selectively isolated and well preserved. The lesion was completely excised and sent for histopathological analysis. There was only one instance of temporary bradycardia throughout the procedure. Patient made an uneventful recovery with no lower cranial nerve deficits or any vascular insults. The histology from the lesion revealed a characteristic Zellballen pattern, highly suggestive of a Paraganglioma ( Figure 5). The patient has been for regular follow-ups in the last 4 months, with complete resolution of her previous symptoms. She has been advised for lifelong periodic visits.

## Discussion

Carotid body tumors, first described by Von Haller in 1743, are of neuro-ectodermal origin ^3^. They are the most common tumors of this origin to occur in the head and neck region ^4^. Since the majority of cases seem to occur in people residing at high altitudes, chronic hypoxemia has been postulated to be a cause for such tumors ^5^. Most patients present with features owing to compression of the lower cranial nerves by the lesion ^6^ In few cases, paroxysmal symptoms resulting from excessive circulating catecholamine may be evident ^7, 8^.

Examination should begin by assessing the pulsatility of the tumor. It has a characteristic mobility in the horizontal direction whereas it has restricted mobility in the vertical direction. This aspect is also referred to as the ‘Fontaine sign’ ^9^.

Duplex ultrasound study of the carotid vessels is the first line diagnostic imaging modality ^10^. It helps diagnose the lesion, determine its extent and the involvement of the major vessels. It also aids in simultaneous assessment of both the carotid vessels, to rule out bilateral involvement and carotid artery disease, especially in aged patient groups. In high-risk patients with positive family history or in active catecholamine secreting lesions, this type of imaging can help rule out multiple infections by evaluating the adrenal glands. Shamblin *et al* classified these highly vascular lesions depending on how they are placed in relation to the carotid vessel, which helps plan their management strategy ^11^. Most high-grade lesions require an adjuvant protocol, either in the form of preoperative embolisation or intra-operative temporary or permanent interposition vascular grafts.Needle biopsy is contraindicated due to the risk of haemorrhage, thrombosis or pseudo-aneurysm ^12^. It is also advisable to rule out synchronous neural crest lesions by imaging the tympanic cavity and the adrenal glands, especially in patients with positive family history ^13^. Although rare, active lesions (seen in around 5% of cases) should be ruled out by assessing the plasma catecholamine or the urinary VMA levels ^14^.

The risk of intra-operative and post-operative neurological and the vascular complications increases with the grade of the lesions. The size, vascularity and thereby intra-operative blood loss is minimized with the use of embolisation ^15^. However, there is an inadvertent risk of stroke and increased difficulty in excising the tumor, when it is glued with embolized particles intra-operatively ^16^.

The first successful excision of a carotid body tumor was performed by Albert J Van Der Kogel in 1889 ^17^. Surgical excision is the management modality of choice, with embolisation and radiotherapy used as adjuncts in a few selected cases ^10^. Cerebral protection from hypotensive insults during vascular clamping or bypass and management of arrhythmias (following vagal nerve stimulation) during tumor manipulation are some of the challenges faced during the intra-operative period ^18, 19^. Surgery may be carried out under local, regional or general anesthesia. Local or cervical plexus block offers the advantage of a continuous neurological assessment to rule out cerebral hypo-perfusion ^20, 21^. Blood loss can be minimized with meticulous sub-adventitial dissection of the lesion from the vessel walls. Intra-operative vascular control can be achieved by temporary carotid ligation, use of temporary arterial bypass or by use of vascular inter-position graft in cases of inadvertent tear within the vessels ^22^. Hypotensive anesthesia is another valid option to maintain a bloodless field ^23^. A temporary clamp of less than 10 minutes is considered safe ^13^. The IJV, vagus nerve and hypoglossal nerve should always be isolated and well protected throughout the procedure. The higher the grade of the lesion and the need for repair or reconstruction, the higher the risk of stroke ^24, 25^.

Huge carotid body paragangliomas continue to cause a high incidence of pre- and postoperative complications ^17, 26^, including peri-operative stroke and persistent nerve palsy ^27^.

Cranial nerve palsy is seen in as high as 40% of cases ^28^. The hypoglossal nerve is the most common nerve to be affected in the post-operative period, thereby undue stretching should be avoided. Mortality has been seen in 3% of cases, with tumors greater than 5 cm ^29^. In cases of transmural involvement of the vessel by the tumor, excision of the main vessel along with the tumor is justified. Internal carotid artery ligation with reconstruction requirements as well as permanent cranial nerve deficits have been observed in 23% of cases, all belonging to Shambling grade 3 tumors ^30^.

A characteristic Zellballen pattern in the histology and positive staining with neuron-specific enolase (NSE) for Paraganglioma and S100 for sustentacular cells is definitive in diagnosing the tumor ^31^.

There are no defined pathological criteria to differentiate benign tumors from their malignant counterpart. Distant metastasis and the involvement of the lymph nodes are the only definitive markers to determine its malignant behavior ^32^.

Malignant transformation and local or distant metastasis is estimated to affect around 10% of cases ^33^. Follow-up with doppler and duplex ultrasound of the carotid vessels is currently advocated ^17^.

 Lifelong follow-ups are required ^34^. Succinate dehydrogenase (SDH) mutations are commonly seen in young patients, and testing for these may provide us with clues when assessing patients at high risk ^35^.

## Conclusions

Large and complicated vascular tumors can be managed with proper planning and execution ^36^. With judicious anesthetic care and meticulous subadventitial dissection, such lesions can be managed even in a rural setup ^37^.

## Consent

Written informed consent for publication of clinical data and clinical images was obtained from the patient.
