# Supplementary material for: Case Report: Managing a giant, high-grade carotid body tumor in a resource-limited setting
Source: F1000Res. 2017 Oct 4;6:1801. [Version 1] doi: 10.12688/f1000research.12726.1 (PMC5717474; doi:10.12688/f1000research.12726.1)
Supplement: Peer review history

# Peer review: Referee response for version 1

**DOI:** 10.5256/f1000research.13784.r28491

**Published:** 2017-12-11

**Authors:** Guru Dutta Satyarthee

**Affiliations:** 1 Department of Neurosurgery, Neurosciences Centre, All India Institute of Medical Sciences (AIIMS), Gamma Knife Centre, New Delhi, Delhi, India

Paragangliomas are neoplasms arising from extra-adrenal chromaffin tissue. It frequently cause symptoms by overproduction of catecholamines and carry risk of hypertension during surgery and post –excision, patient may develop hemodynamic insatiability [1]. Carotid body tumor is a slow-growing neoplasm, usually localized at the bifurcation of the common carotid artery represent most commonly encountered are head and neck paragangliomas, accounting for 60–78% [2]. It is usually benign and rarely malignant and accounts for about 0.5% of all head and neck tumors [2].

 In 1971,  Shamblin *et al*. classified carotid body tumor into three classes: in Class I tumor is small and surgically resectable, Class II includes adherent tumor, which surrounding the carotid vessels partially, resection is considered as technically more difficult, and class III, the  size of  the tumor is large ie more than 5 cm and usually completely encases the carotid vessels, resection is considered very difficult, and always carry risk of the cerebral circulation interruption always [3].

 DSA is considered as important imaging modality of choice for diagnosis as clearly defines, epicentre of tumor, location, size and encasement of vessel branches in tumor and aid in aiding transarterial embolization therapy which reduces intraoperative blood loss. However, it carry limitation poor visualization of adherence of the tumor mass to the peripheral tissues, cerebral embolism causing ischemic stroke and vascular injury are also reported and even risk cardiac asystole with carotid body hypersensitivity. However DSA of cerebral vessels including cross circulation is essential if needed of cerebral vessel revascularization surgery is required [4]. So, currently, computed tomography scan, CT angiography and MRI are preffered routinely .  

 Surgery is preferred treatment modality and chief concern s include highly vascular mass, dense adherence to the vagus nerve. Hua *et al* observed microneurosurgical technique is said in dissection. And prevent vascular and cranial nerve injury [1]. Radiotherapy is an alternative management option for patients with co-morbid illness or who may not tolerate the surgery, presence of  metastasis subsequent to surgery complications include otitis externa, otitis media, osteoradionecrosis, cranial nerve lesion and brain injury [5].  Munakomi  and neuroanesthesia team should be commended for successful  surgery, perioperative hemodynamic adequate control  with good outcome.

I have read this submission. I believe that I have an appropriate level of expertise to confirm that it is of an acceptable scientific standard.

## References

[1] HuaQXuZJiangY: Diagnosis and surgical treatment of carotid body tumor: A retrospective analysis of 58 patients. *Oncol Lett*.2017;14(3) : 10.3892/ol.2017.6582 3628-3632 10.3892/ol.2017.6582 28927123PMC5587999

[2] KollertMMinoviAADrafWBockmühlU: Cervical paragangliomas-tumor control and long-term functional results after surgery. *Skull Base*.2006;16(4) : 10.1055/s-2006-950386 185-91 10.1055/s-2006-950386 17471317PMC1766458

[3] ShamblinWRReMineWHShepsSGHarrisonEG: Carotid body tumor (chemodectoma). Clinicopathologic analysis of ninety cases. *Am J Surg*.1971;122(6) :732-9 512772410.1016/0002-9610(71)90436-3

[4] PowerAHBowerTCKasperbauerJLinkMJOderichGCloftHYoungWFGloviczkiP: Impact of preoperative embolization on outcomes of carotid body tumor resections. *J Vasc Surg*.2012;56(4) : 10.1016/j.jvs.2012.03.037 979-89 10.1016/j.jvs.2012.03.037 22727841

[5] KünzelJKochMBraseCFietkauRIroHZenkJ: Treatment of cervical paragangliomas: is surgery the only way?. *Am J Otolaryngol*.35(2) : 10.1016/j.amjoto.2013.08.020 186-91 10.1016/j.amjoto.2013.08.020 24051237

# Peer review: Referee response for version 1

**DOI:** 10.5256/f1000research.13784.r28506

**Published:** 2017-12-01

**Authors:** Luis Rafael Moscote-Salazar, Alexis Narvaez-Rojas

**Affiliations:** 1 Department of Neurosurgery, RED LATINO (Latin American Trauma & Intensive Neurocare Organization), Bogotá, Colombia; 2 University of Cartagena, Cartagena, Colombia; 3 Universidad Nacional Autónoma de Nicaragua, Managua, Nicaragua

Paragangliomas are a type of infrequent head and neck tumor originating in the paraganglionic brachymeric tissue involved in the development of the branchial arches. Five families of paragangliomas have been described:

 Brachymeric, intravagal, sympathetic, viscero-autonomic and adrenal. At the histopathological level, this type of tumors has an identical configuration regardless of its origin. Sporadic cases of this class of tumors are more frequent in females. A high frequency of this class of tumors has been established in patients who live at high altitudes and those suffering from the chronic obstructive disease. It has been suggested that chronic hypoxemia plays a role in the genesis of this class of tumors

 Munakomi *et al.* presented a very well documented case and with successful resolution managed in a resource-limited setting.

 The therapeutic choice depends fundamentally on the location of this kind of tumors, the size of the lesion, age, health status of the patient plus the experience of the surgeons. The use of various resources as angiography and preoperative embolization suggests that this type of tumors should be ideally managed in third-level care centers. The case managed by Munakomi *et al.* testifies that the preparation and expertise of the surgeon are fundamental for the successful resolution of this class of injuries.

 Finally, it is important to mention that this type of tumors has a significant risk of urinary complications and several postoperative complications have been described, including cranial nerve lesions, cerebral infarction, hemorrhages among others.

We have read this submission. We believe that we have an appropriate level of expertise to confirm that it is of an acceptable scientific standard.

# Peer review: Referee response for version 1

**DOI:** 10.5256/f1000research.13784.r27906

**Published:** 2017-11-24

**Authors:** Yi Guo

**Affiliations:** 1 Department of Neurosurgery, Tsinghua Changgung Hospital, Beijing, China

This is a well-written case report about how to manage a Shamblin Grade 3 carotid body tumor (CBT). Though surgery is first-line treatment of CBT, it remains a challenge because it’s highly vascular and adherent to the vagus nerve in high-grade CBT. The authors discussed the diagnosis and treatment of CBT in detail. In large tumors like this case, cerebral collateral circulation should be evaluated in case of vessel transplantation or ligation performed. HyperForm might be used for intra-operative distal vascular control if tumor is very large to reach the skull base. However, in a resource-limited setting it is difficult for detailed pre-operative evaluation and intra-operative vascular control.

I have read this submission. I believe that I have an appropriate level of expertise to confirm that it is of an acceptable scientific standard.

# Peer review: Referee response for version 1

**DOI:** 10.5256/f1000research.13784.r26639

**Published:** 2017-11-13

**Authors:** Arif Hussian Sarmast

**Affiliations:** 1 Department of Neurosurgery, Jawaharlal Nehru Medical College, Aligarh Muslim University, Aligarh Uttar Pradesh, India

It is a very beautifully written case report, however I would like to clarify from the authors how a giant carotid body tumour is to be addressed in a normal setting, resource limitation does not change the management in a big chunk of cases like that. It needs a change in headline.

I have read this submission. I believe that I have an appropriate level of expertise to confirm that it is of an acceptable scientific standard, however I have significant reservations, as outlined above.
